# Advances in Infectious Diseases and Clinical Microbiology during the COVID-19 Pandemic

**DOI:** 10.3390/medicina58101362

**Published:** 2022-09-28

**Authors:** Yusra Habib Khan, Tauqeer Hussain Mallhi, Tahir Mehmood Khan, Muhammad Salman

**Affiliations:** 1 Department of Clinical Pharmacy, College of Pharmacy, Jouf University, Sakaka 72341, Saudi Arabia; 2 Institute of Pharmaceutical Sciences, University of Veterinary & Animal Sciences, Lahore 54000, Pakistan; 3 Institute of Pharmacy, Faculty of Pharmaceutical and Allied Health Sciences, Lahore College for Women University, Lahore 54000, Pakistan

Infectious diseases pose substantial challenges to the healthcare system and are associated with significant morbidity and mortality. The COVID-19 pandemic has necessitated considerable public health maneuvers across the globe [1]. Changes in the patterns of various infectious diseases were observed during the pandemic. A growing body of evidence indicates the decline in influenza virus circulation during the COVID-19 pandemic in various regions around the world that is primarily related to several controlling measures and non-pharmaceutical interventions employed to curb the growing encumbrance of SARS-CoV-2 [2]. However, the COVID-19 pandemic has caused disruptions in reporting of other infectious diseases, such as dengue, sexually transmitted diseases, and other coronavirus infections [3]. Likewise, this pandemic has also disrupted various routine immunization campaigns [4]. Other infections such as HIV/AIDS, tuberculosis, and hepatitis carry equivalent health risks to the global population. The reduction in the reporting of these infections due to an overwhelmed healthcare system during the COVID-19 pandemic remained a significant concern for health authorities. Understanding the transmission patterns of other infectious diseases during the COVID-19 pandemic is of utmost importance for designing and implementing mitigating strategies for public health. However, there is a dearth of investigations on this topic. Ascertaining the impact of the COVID-19 pandemic on the incidence of other infectious diseases will improve the responsiveness of health authorities during disease outbreaks in future. It is pertinent to mention that the COVID-19 pandemic also resulted in improved healthcare metrics for other infectious diseases, i.e., improved influenza vaccination rate during the pandemic. On the other hand, the implications, scope, and relevance of clinical microbiology have gained invaluable appreciation during this pandemic [5]. Clinical microbiologists played an aggressive role during the pandemic through the provision of various expert services such as the identification of viral structure, differential diagnosis, and optimized sterilization and disinfection processes. The COVID-19 pandemic has initiated new horizons for infectious disease research, control, funding acquisitions, administrative assistance, and health promotion. This issue emphasizes various aspects of the advancements and progress in infectious diseases and clinical microbiology during the ongoing COVID-19 pandemic. Potential topics include, but are not limited to, the following:

- Impact of the COVID-19 pandemic on the reporting of infectious diseases;
- Emerging atypical complications during the disease course of COVID-19;
- Clinical and laboratory characteristics and outcomes among COVID-19 patients with co-infections;
- The burden of common and major infectious diseases before, during, and after the COVID-19 pandemic;
- Challenges in the clinical management of serious and neglected tropical infections;
- Impact of the COVID-19 pandemic on the development and repurposing of drugs against SARS-CoV-2;
- Changes in vaccination patterns for other infectious diseases during the COVID-19 pandemic;
- Correlation of COVID-19 vaccination campaigns with routine immunization programs;
- Challenges in the management of infectious diseases during the COVID-19 pandemic;
- Disruptions in preventive measures or services for vaccine-preventable diseases (VPDs) during the COVID-19 pandemic;
- Evolution of Scope of Clinical Microbiology in response to the rapid spread of COVID-19;
- Implications of knowledge related to Clinical Microbiology in the identification and screening of SARS-CoV-2;
- Variations in response of health authorities to the challenges during the pandemic, i.e., conspiracy beliefs or theories, control measures and mandates;
- Epidemiological variations in infectious diseases during the COVID-19 pandemic, more specifically during the period of lockdowns;
- Implications of information technology for screening, identification, and control of SARS-CoV-2.

This Special Issue welcomes submissions ranging from original, clinical, and translational articles to reviews in the field of Clinical Microbiology and Infectious Diseases during the COVID-19 Pandemic.

## Data Availability

Not applicable.

## References

[1] Amar S., Avni Y.S., O’Rourke N., Michael T. (2022). Prevalence of common infectious diseases after COVID-19 vaccination and easing of pandemic restrictions in Israel. JAMA Netw. Open.

[2] Khanolkar R.A., Trajkovski A., Agarwal A., Pauls M.A., Lang E.S. (2022). Emerging evidence for non-pharmacologic interventions in reducing the burden of respiratory illnesses. Intern. Emerg. Med..

[3] Chen Y., Li N., Lourenço J., Wang L., Cazelles B., Dong L., Li B., Liu Y., Jit M., Bosse N.I. (2022). Measuring the effects of COVID-19-related disruption on dengue transmission in southeast Asia and Latin America: A statistical modelling study. Lancet Infect. Dis..

[4] Ota M.O.C., Badur S., Romano-Mazzotti L., Friedland L.R. (2021). Impact of COVID-19 pandemic on routine immunization. Ann. Med..

[5] Dunbar S., Babady E., Das S., Moore C. (2022). Impact of COVID-19 on the clinical microbiology laboratory: Preparing for the next pandemic. Front. Cell. Infect. Microbiol..

